# Cyanines Substituted on the Polymethine Chain: Synthesis, Resulting Properties, and Application Use Cases

**DOI:** 10.1002/cplu.202500279

**Published:** 2025-10-03

**Authors:** Rebecca Strada, David Dunlop, Peter Šebej

**Affiliations:** ^1^ RECETOX Faculty of Science Masaryk University Kamenice 5 61137 Brno Czech Republic; ^2^ Institute of Organic Chemistry and Biochemistry of the Czech Academy of Sciences Flemingovo náměstí 542/2 16000 Prague 6 Czech Republic; ^3^ Department of Inorganic Chemistry Faculty of Science Charles University in Prague Hlavova 2030 12840 Prague 2 Czech Republic

**Keywords:** applications, cyanines, dyes, polymethines, spectroscopy, structure–property relationships, substituents

## Abstract

Cyanines comprise a diverse group of small‐molecule polymethine dyes combining tunable optical properties with high molar absorptivity and fluorescence emission quantum yield, enabling various applications in bioimaging, diagnostics, molecular electronics, photonics, and nonlinear optics. These applications can be facilitated by adjusting the length of their polymethine chain and their functionalization through their end groups or the polymethine chain. Yet, the latter approach remains largely unexplored, with limited information scattered throughout literature. This review focuses on cyanines substituted on their chain, covering their synthesis, properties, and applications and providing an overview of how substituents on their polymethine chain influences their spectroscopic properties, akin to other factors, such as polymethine length and end groups. Lastly, this review illustrates how substituents on the polymethine chain facilitate the application of cyanine dyes in promising research areas.

## Graphical abstract

Cyanines are small‐molecule dyes with a polymethine chain. For various applications, their properties can be tuned by varying not only chain length and auxochrome end groups but also the substituents on the polymethine chain. Here, we review the synthesis, properties, and applications of cyanines with substituents on the polymethine chain.

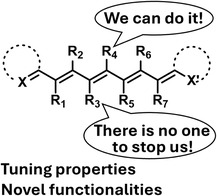

## 1 Introduction

**Figure 1:**
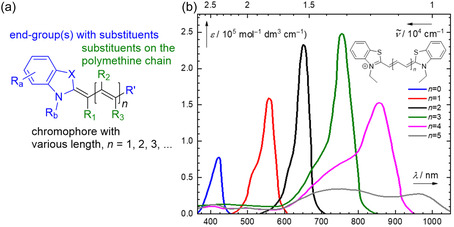
a) General structure of a cyanine dye—chromophore with auxochromes: end groups (R’; the indolenine end group typical of the cyanine subfamily is shown as an example on the other side with further functionalization options (R_a_–R_b_)) and substituents on the polymethine chain (R_1_–R_*n*_). b) Absorption spectra of typical cyanines: *n* = 0, monomethine; *n* = 1, trimethine; *n* = 2, pentamethine; *n* = 3, heptamethine, and *n* = 4, nonamethine cyanines. Reproduced with permission.^[^ ^140^ ^]^ Copyright 2017, IOP Publishing Ltd.

Cyanines are a large family of small‐molecule organic dyes. Thebackbone ofthese dyes, their *chromophore*, is a polymethine chain, i.e., a conjugated hydrocarbon chain with an odd number of methine groups (**Figure** **1**). This polymethine chain is appended by *auxochrome* moieties, typically *N*‐heterocyclic.

The photophysical properties of cyanine dyes are determined by three structural features: 1) polymethine chain length, 2) end group(s) and their substituents, and 3) substituents on the polymethine chain (Figure 1a). Polymethine chain length has the strongest effect on absorption and emission maxima of cyanines, which are usually found around 500–550 nm for trimethine,^[^ ^1^ ^]^ 625–675 nm for pentamethine,^[^ ^2^ ^]^ 725–850 nm for heptamethine,^[^ ^3^ ^,^ ^4^ ^]^ and >850 nm for nonamethine^[^ ^5^ ^]^ cyanines (Figure 1b).^[^ ^6^ ^]^

Heptamethine cyanines, in particular, display absorption and emission bands with maxima in the red and near infra‐red (NIR) spectral region, where absorption is particularly low in mammal tissues.^[^ ^7^ ^,^ ^8^ ^]^ This spectral range around 650–950 nm is also known as the “tissue‐transparent window” (ttw),^[^ ^9^ ^]^ the first of a few such spectral ranges in the infrared (IR) region.^[^ ^10^ ^,^ ^11^ ^]^ For this reason, research interest in heptamethines, nonamethines,^[^ ^12^ ^,^ ^13^ ^]^ and longer polymethine cyanines^[^ ^5^ ^]^ has been increasing in recent years.

These longer cyanines, however, suffer from lower stability, which decreases with chain length. Their characterization is also hindered by the limited availability of sensitive optical detectors in the short‐wavelength infra‐red (SWIR) spectral region.^[^ ^14^ ^]^ Consequently, multiple undecamethine cyanines have been incompletely characterized,^[^ ^15^ ^]^ most controversially Soviet dye No. 3955 with *λ*_abs_(max) ≈ 1010 nm,^[^ ^16^ ^]^ which was later disputed as a two‐species mixture.^[^ ^17^ ^]^

Moreover, longer cyanines (e.g., undecamethines, *n* = 5, Figure 1b) tend to mimic the spectral properties of short cyanines due to symmetry breaking and distortion in their electron density distribution.^[^ ^18^ ^]^ So while increasing the length of cyanines may enable us to extend absorption further into the IR region, their structural instability and complex spectral properties continue to pose major challenges for their characterization and applications.

**Figure 2:**
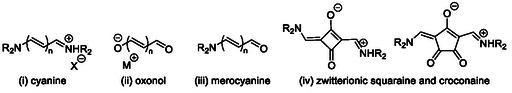
Classification of cyanine dyes based on their molecular charge: i) cationic cyanines (covered in this review), ii) anionic oxonols, iii) neutral merocyanines, and iv) zwitterionic squaraines and croconaines.

**Figure 3:**
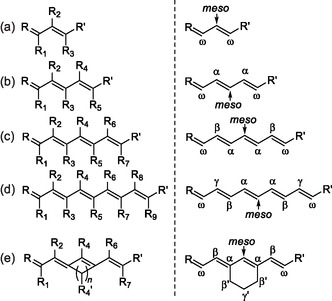
Structures, numbering, and nomenclature of cyanines with different polymethine chain lengths: a) trimethines, b) pentamethines, c) heptamethines, d) nonamethines, and e) heptamethine with an embedded carbocycle (an example). Left column: numbering of substituents; and right column: nomenclature of positions used throughout this paper to highlight the role of the symmetry of polymethine chains with an odd number of carbon units.

The end groups of cyanine dyes, typically heterocyclic moieties such as pyrrole, imidazole, thiazole, pyridine, quinoline, indole, and benzothiazole, also modulate their spectroscopic properties. Cyanines symmetrically functionalized with the same heterocyclic end groups (R = R’; **Figure** **2**, **3**) are the most synthetically accessible. But polymethine chains can also be functionalized nonsymmetrically, which can influence their charge, thereby yielding the following classes of polymethine dyes: i) cationic cyanines, the topic of this review; ii) anionic oxonols; iii) neutral merocyanines; and iv) zwitterionic polymethine dyes, including squaraines^[^ ^19^ ^]^ and croconaines^[^ ^20^ ^,^ ^21^ ^]^ (Figure 2).^[^ ^22^ ^]^ Although some of these classes of polymethine dyes are sometimes colloquially referred to as cyanines, we apply these distinctions consistently, covering only cationic, polymethine cyanines.

The two most common design principles, namely 1) extending polymethine chain length and 2) end‐group substitution, have been addressed in previous reviews,^[^ ^23^, ^24^, ^25^, ^26^, ^27^ ^–^ ^28^ ^]^ including their applications in imaging,^[^ ^27^ ^,^ ^28^ ^]^ photodynamic therapy (PDT),^[^ ^29^ ^]^ chemosensing,^[^ ^30^ ^]^ and photocages^[^ ^31^ ^]^ such as anticancer drugs.^[^ ^32^ ^]^ By contrast, in this review, we highlight the most overlooked structural feature: substituents on the polymethine chain. Focusing on this underexplored dimension, we provide the first comprehensive review of cyanines substituted on the polymethine chain, showcasing available synthetic approaches to such cyanines. We also shed light on their spectral properties before presenting current and potential applications of these cyanines.

To facilitate comparisons between cyanines with a different number of C(H or X) units in the polymethine chain and to emphasize the role of symmetry in many of them, we do not follow the IUPAC numbering of R_*n*_ with *n* = 1, 2, 3… in this study. Instead, carbon atoms are numbered from the middle position (*meso*‐, Figure 3). Then, neighboring carbons are denoted with a Greek letter, namely *α*, *β*, … *ω*, prioritizing the notation that shows the position in the polymethine, e.g., the middle *meso*‐position (Figure 3).

## 2 Synthesis of Polymethine Cyanines Substituted at the *Meso*‐ and/or *α‐*Position

**Scheme 1:**
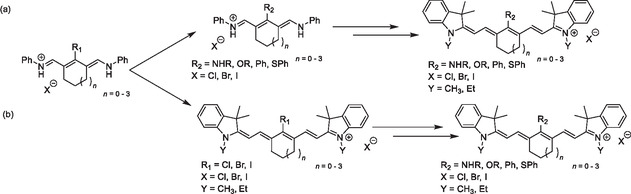
Two approaches to the synthesis of cyanines with functional group(s) on their polymethine chain (with examples of heptamethine cyanines substituted at the *meso‐*position): a) the functional groups are introduced to the polymethine chain precursor (top row), and b) the functional groups are introduced or exchanged on the polymethine chain (bottom row). Note: the substituents rank among the most common examples but do not compose an exhaustive list.

Cyanines are typically synthesized through the condensation of heterocyclic compounds (end‐group precursors) with a suitable polymethine chain precursor in an alcohol solvent, optionally with a base catalyst.^[^ ^33^, ^34^ ^–^ ^35^ ^]^ Current approaches to chain‐substituted cyanines can be divided into two groups: a) introducing substituent(s) to the polymethine chain precursor *before the condensation reaction* (**Scheme** **1**a),^[^ ^34^ ^]^ and b) introducing or modifying substituent(s) on the polymethine chain *after the condensation reaction* (Scheme 1b).^[^ ^36^ ^]^ These methods are used to prepare cyanines bearing substituents at the *meso‐*position or adjacent, *α‐*, positions. In the latter, the *α‐*positions are typically linked through five‐ or six‐membered, carbocyclic or heterocyclic rings.^[^ ^36^ ^,^ ^37^ ^]^

### 2.1 Introducing Substituent(s) to Cyanine Polymethine Chain Precursors before Their Condensation

**Scheme 2:**
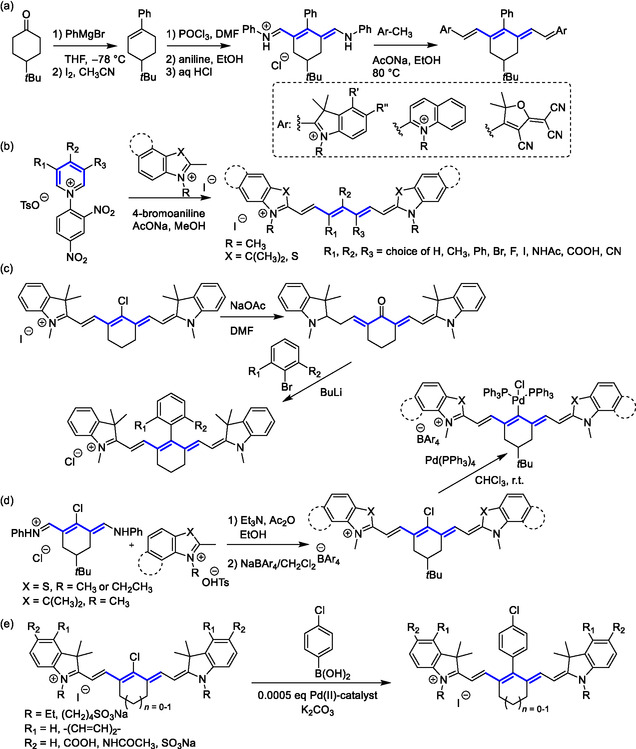
Reaction pathways leading to heptamethine cyanines with substituents at the *α*‐, *meso*‐ and *α*‐positions. Condensation of a heterocyclic end‐group precursor with an a) acyclic and a b) cyclic pentamethine unit (Zincke salt) with substituents.^[^ ^40^ ^]^ Functional group(s) interconversion at the *meso*‐position: c) nucleophilic attack of in situ prepared aryllithium on the keto‐form of *meso*‐hydroxy cyanine,^[^ ^3^ ^]^ d) Pd‐functionalized heptamethine preparation,^[^ ^53^ ^]^ and e) organopalladium reagent‐facilitated Suzuki–Miyaura cross‐coupling.^[^ ^56^ ^]^

**Scheme 3:**
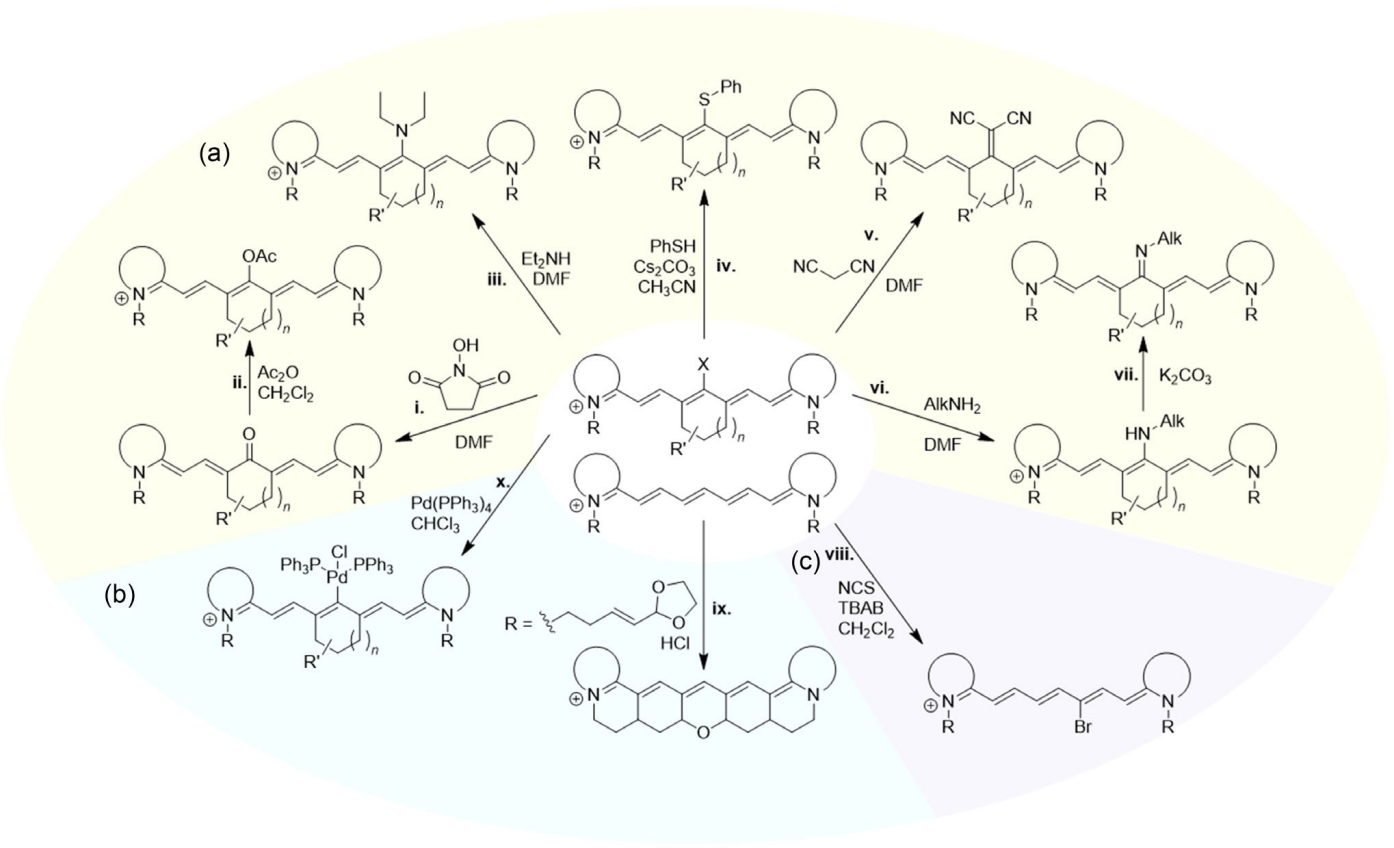
Overview of reactions yielding structurally diverse cyanines with substituted polymethine chains via introduction or exchange of functional groups on the polymethine chain by a) nucleophilic substitution (highlighted in yellow), pathways i–vii;^[^ ^42^ ^]^ b) by electrophilic substitution (highlighted in violet), pathway viii;^[^ ^58^ ^]^ and c) other reactions (highlighted in blue), pathways ix^[^ ^63^ ^]^ and x.^[^ ^56^ ^]^

In the first approach, substituents are introduced to the polymethine chain precursor *before the condensation reaction* (Scheme 1a). Following this approach, heptamethine cyanines aryl‐substituted at the *meso*‐position^[^ ^38^ ^]^ can be prepared *in sequence*, starting with a commercially available cyclohexanone derivative. To this derivative, the intended *meso*‐ substituent is introduced via, e.g., a Grignard reaction, prior to a double Vilsmeier–Haack reaction and Schiff base formation, which yield the linear, *meso*‐substituted polymethine precursor (**Scheme** **2**a). Upon condensation with end‐group precursors, this linear precursor then leads to the target cyanine.^[^ ^39^ ^]^ This approach is straightforward but limits the variety of *α*‐substituents (**Scheme** **3**, i, ii, and iii).

In contrast, a large scope of various *meso*‐ and *α*‐substituent(s) can be inherited from a cyclic precursor in a modified version of the Zincke reaction. In this reaction, the end‐group precursor heterocycle nucleophilically attacks the pyridinium Zincke salt, yielding a heptamethine chain from the five carbons of the pyridinium ring upon its opening (Scheme 2b),^[^ ^40^ ^]^ as in the opening of a 2,6‐dialkyloxy‐dihydropyran.^[^ ^41^ ^]^ This approach offers a complementary route to heptamethine cyanines, whereby key substituents are retained while leveraging the Zincke reaction for efficient chain assembly.

### 2.2 Introducing or Modifying Substituent(s) on the Cyanine Polymethine Chain after Condensation

In the second approach, the substituent is introduced *after the condensation reaction* (Scheme 1b). This approach is equally versatile in functional group interconversion at the *meso*‐position. For example, halogens, which are good leaving groups, undergo efficient substitution by various nucleophiles, including amines, amino acids, thiols, and aromatic or aliphatic alcohols (Scheme 3, iii, iv, vi).^[^ ^42^, ^43^, ^44^ ^–^ ^45^ ^]^ Substitution often proceeds via an S_*N*_1‐type mechanism facilitated by electron transfer from the nucleophile to the cationic cyanine.^[^ ^46^ ^]^ Aprotic polar solvents, such as dimethylformamide (DMF) and dimethylsulfoxide, further promote this reaction by maintaining a stable concentration of radical anion intermediates.^[^ ^47^ ^]^

In an iteration of this approach, the *meso*‐position can be oxidized during which its substituent, acting as a leaving group, is exchanged for an oxygen atom, yielding a carbonyl (Scheme 3, i). At the middle (*meso*‐) position of a heptamethine cyanine, the carbonyl group hinders its polymethine chain conjugation. As a result, such polymethine also becomes a fluorescent pH sensor driven by keto‐enol tautomerism, whereby the enol form restores conjugation, with substantially redshifted absorption and emission spectra.^[^ ^48^ ^]^ This equilibrium is also temperature sensitive.^[^ ^49^ ^]^ Upon nucleophilic addition of aryllithium reagents, the keto‐form of oxidized heptamethine acts as a key intermediate in late‐stage arylation of heptamethines (Scheme 2c).^[^ ^3^ ^]^ Similar nucleophilic substitution at the *meso‐*position is commonly used to introduce linkers and groups for conjugation, targeting ligands, and bio(macro)molecules.^[^ ^50^ ^,^ ^51^ ^]^

Other functional group interconversions known in cyanines include Smiles rearrangement and carbon–carbon coupling. Smiles rearrangement provides access to *meso*‐*O*‐alkyl heptamethine cyanines otherwise inaccessible through direct *meso*‐halogen substitution.^[^ ^52^ ^]^ Through Suzuki and Sonogashira coupling reactions, aryls and ethynyls, respectively, can be introduced at the *meso*‐position.^[^ ^50^ ^]^ For instance, *meso*‐Cl‐substituted heptamethine cyanine undergoes oxidative addition to Pd(PPh_3_)_4_, under an inert atmosphere (Scheme 2d, Scheme 3, x).^[^ ^53^ ^]^ Accordingly, the chlorine atom at the *meso‐*position can be substituted by the aryl moiety via Pd‐catalyzed^[^ ^54^ ^,^ ^55^ ^]^ Suzuki–Miyaura coupling, as exemplified in Scheme 2e.^[^ ^56^ ^]^

**Scheme 4:**
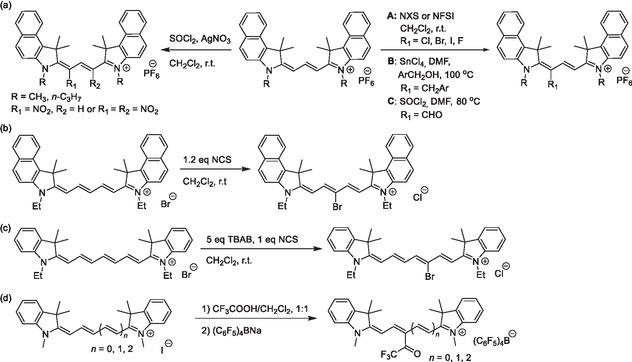
Electrophilic substitutions on the polymethine chain of cyanines with various electrophiles: a) trimethine, b) pentamethine, c) heptamethine halogenation, and d) trifluoroacetylation.

Polymethine chains are also prone to electrophilic substitutions (Scheme 3, viii). In cyanines, though, these electrophilic substitutions show length‐dependent site selectivity. In trimethines and heptamethines, the *α*‐position undergoes electrophilic substitution (**Scheme** **4**a,c).^[^ ^57^ ^]^ In pentamethines, conversely, the *meso*‐position undergoes electrophilic substitution (Scheme 4b).^[^ ^58^ ^]^

Hydrogen atoms of the polymethine chain can be substituted by the (pseudo)halide counterion of the cyanine using *N*‐chlorosuccinimide (NCS). In this approach, NCS forms an inter(pseudo)halogen intermediate with the cyanine counterion, thereby facilitating electrophilic substitution (Scheme 4a–c).^[^ ^58^ ^]^ The counterion of the cyanine is then replaced by a chloride anion. However, using halogenation reagents with a redox potential lower than that of NCS, such as *N*‐iodosuccinimide (NIS) or *N*‐bromosuccinimide (NBS),^[^ ^59^ ^,^ ^60^ ^]^ instead results in direct halogenation of the cyanine.^[^ ^58^ ^]^

Electrophilic substitution of cyanines is not limited to (pseudo)halides. Trimethines undergo electrophilic, Lewis‐acid‐catalyzed alkylations at the *α*‐position.^[^ ^57^ ^]^ In DMF and SOCl_2_, the formyl group has also been introduced at the same position (Scheme 4a).^[^ ^57^ ^]^ Furthermore, both *α*‐positions of trimethines (where *α*‐ is also *ω*‐position) have been nitrated using mild nitration agents, such as nitrosyl chloride generated in situ from a mixture of AgNO_3_/SOCl_2_ (Scheme 4a).^[^ ^57^ ^]^ The hydrogen in the electron‐rich position of polymethine cyanines (*α*‐/*ω*‐ in trimethine, *meso*‐ in pentamethine, and *α*‐ in heptamethine) can also be easily exchanged for a trifluoromethyl in a similar electrophilic reaction (Scheme 4d).^[^ ^61^ ^]^

### 2.3 Synthesis of Cyanines Substituted at *β*‐ and *ω*‐Positions and Multisubstituted Cyanines

Cyanines substituted at positions other than *meso*‐ and *α‐* can be prepared using the same two approaches, namely condensation of a heterocycle precursor with a chain precursor bearing the substituent or substitution on the polymethine chain after the condensation reaction (Scheme 1). In addition, the substituents can also be introduced to end‐group heterocycle precursors, leading to *ω*‐substituted cyanine. However, the literature on cyanines substituted at *β*‐ and *ω*‐positions remains scarce. Therefore, in this chapter, we provide a brief overview of some examples on a case‐by‐case basis.

#### 2.3.1 Substituents at *β*‐Positions, Next to Terminal Positions (R_2_, R_6_ for Heptamethines)

**Scheme 5:**
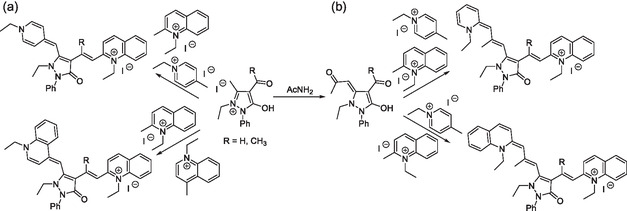
Microwave‐assisted, base‐catalyzed condensation of substituted pyrazole iodide with nitrogen heterocycle end‐group precursors yields a) pentamethine cyanine analogs with substituents at one or both *α‐*positions (left), and b) heptamethine cyanine analogs with alkyl substituted at one or both *β‐*positions (right) (1 mmol; piperidine catalyst; all steps shown above were performed under MW irradiation (200 W)).^[^ ^62^ ^]^

Heptamethine cyanine analogs with substituents at both *β‐*positions of the polymethine chain have been prepared by condensation of a substituted pyrazole iodide and end‐group precursor heterocycles, such as pyridinium and quinolinium salts, over a base catalyst and under microwave irradiation (**Scheme** **5**).^[^ ^62^ ^]^

#### 2.3.2 Substituents at *ω*‐Positions (R_1_, R_7_ for Heptamethines)

**Scheme 6:**
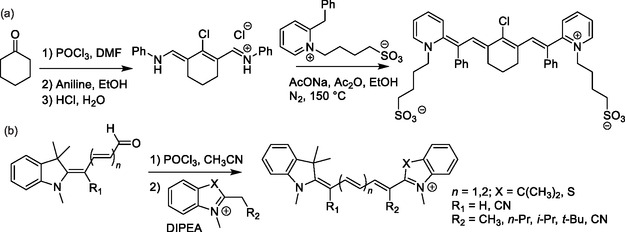
Preparation of heptamethine cyanines substituted at one or both *ω*‐positions (R_1_ and R_7_ of Figure 3c): a) introduction of end‐group heterocycles to a polymethine chain precursor; and b) addition of an end‐group heterocycle to a merocyanine precursor.

The typical condensation of 1,5‐diamino‐pentamethine with cyanine end‐group precursors, usually 2‐alkyl‐*N*‐heterocycles, yields a heptamethine cyanine. In this heptamethine cyanine, the first and seventh carbon atoms derive from the end‐group precursor and can be used to prepare heptamethines with decorated *ω*‐positions, such as 1,7‐diphenyl‐heptamethine (**Scheme** **6**). These polymethine cyanine derivatives hold potential for applications as fluorescent probes^[^ ^62^ ^]^ and photocatalysts.^[^ ^63^ ^]^

Heptamethine cyanine with aryl‐substituted *ω*‐positions can also be prepared in a stepwise fashion using 2‐phenylmethylpyridinium as an end‐group precursor.^[^ ^63^ ^]^ When introducing distinct *ω*‐substituents through stepwise condensation, the *ω*‐positions in heptamethine and pentamethine cyanines derive from the end‐group precursor. This approach enables condensation with both nonsubstituted and substituted chain precursors and can yield both pentamethine and trimethine cyanines. Moreover, a wide scope of functional groups, including alkyls, aryls, and fluoride, has been introduced to *ω*‐positions using this approach.

More recently, researchers have reported a general approach to the functionalization of *ω*‐positions of both pentamethine and heptamethine cyanines. This approach is based on condensation of a terminal alkenylchloride merocyanine precursor (iii in Figure 2), with the corresponding substituted heterocycle, which acts a Fisher's base (Scheme 6b).^[^ ^64^ ^]^ The resulting pentamethine and heptamethine cyanines can bear electron‐withdrawing (EWG; e.g., CN) or electron‐donating (EDG; e.g., CH_3_) groups, as well as more sterically demanding substituents (e.g., *i*‐Pr and *t*‐Bu).

### 2.4 Cyanines with Multiple Substituents on the Polymethine Chain

**Scheme 7:**
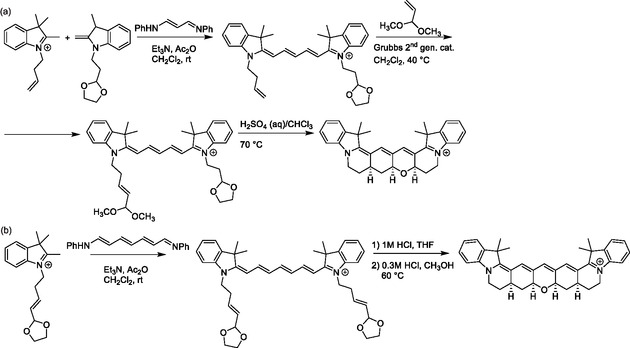
Synthesis of conformationally restricted a) pentamethine and b) heptamethine cyanine dyes.

The synthesis of cyanines with highly substituted chains is particularly challenging and often involves multiple steps. A highly conformationally restricted pentamethine cyanine substituted in both *ω*‐ and *meso*‐positions was prepared through a tetra‐cyclization cascade reaction with a doubly protected dialdehyde precursor (**Scheme** **7**a),^[^ ^65^ ^]^ exemplifying a postmodification of a cyanine chain (Scheme 7a). Using a similar method with a symmetrical, late‐stage heptamethine cyanine precursor, the same research group also prepared an analogous heptamethine cyanine (Scheme 7b).^[^ ^66^ ^]^

**Scheme 8:**
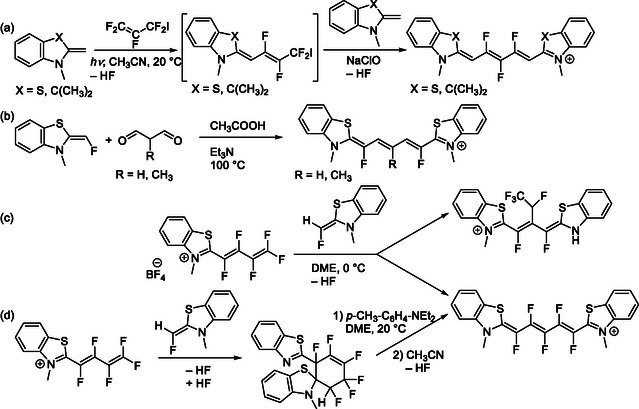
Synthesis of cyanines with fluorine atoms on the chromophore at a) the middle of their polymethine chain (*α*‐ and *meso*‐positions) and b) both positions next to end groups^[^ ^67^ ^]^; c) semicyanine condensation with a perfluorinated polymethine containing a benzothiazole end group, which leads to a mixture of trimethine and pentamethine cyanines; and d) perfluorinated pentamethine cyanine derived from a cyclohexene‐derived intermediate through methylation‐induced ring opening.^[^ ^68^ ^]^

Trimethine cyanines with both *ω*‐positions substituted with EDG (e.g., methyl, methoxy) have been introduced by Yagupolskii and Gruz.^[^ ^67^ ^]^ Conventional approaches have also been applied to synthesize cyanines with a polymethine chain using a polyfluorinated –(CF=CF)_*n*_– precursor and cyclic end‐group intermediates (**Scheme** **8**).^[^ ^68^ ^]^ Such an approach involves a step‐by‐step condensation of 2‐methyl‐benzothiazole with a 3‐iodo‐perfluoro‐propene (Scheme 8a).^[^ ^69^ ^]^ A similar, one‐pot double condensation of 2‐(fluoromethyl)‐benzothiazole with *meso*‐substituted malondialdehyde leads to *ω*,*ω*‐difluoro‐pentamethine cyanine, maintaining the substituent at the *meso*‐position (Scheme 8b).^[^ ^67^ ^]^

Two independent pathways have been followed to synthesize a cyanine with a perfluorinated pentamethine chain (Scheme 8c,d). One pathway involves the sequential addition of thioindolenine end‐group precursors in a step‐by‐step fashion through Stille coupling and condensation. The other, unusual, pathway consists of opening, through HF elimination from a cyclohexene intermediate, with both benzothiazol end groups in place (Scheme 8d).^[^ ^68^ ^]^

The only other known example of a per‐substituted heptamethine is a perdeuterated heptamethine cyanine prepared by opening of a per‐deuterated pyridinium Zincke salt (similarly to Scheme 2b).^[^ ^70^ ^]^ Owing to the heavier deuterium atoms, the perdeuterated heptamethine cyanine has ≈20–30% higher fluorescence emission quantum yields than nondeuterated (all‐H) analogs, which corroborates previous reports of a similar effect on other chromophores upon per‐deuteration of auxochromes^[^ ^71^ ^]^ and regular fluorophores in D_2_O.^[^ ^72^ ^]^

### 2.5 Substituted Polymethine Chains Formed by Cyanine Scission or Dimerization

Changing the polymethine chain length is an unconventional approach to preparing substituted cyanines by exploiting their (photo)degradation pathways. Such photodegradation is often called photoblueing for the blueshift in the main absorption and emission bands of cyanines. This blueshift causes artifacts in super‐resolution microscopy.^[^ ^73^ ^]^

**Scheme 9:**
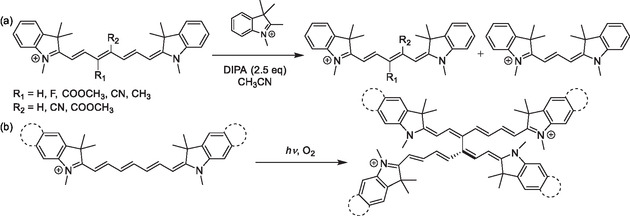
Polymethine chain length can be adjusted by a) thermal truncation of a heptamethine cyanine, which leads to pentamethine and trimethine cyanines, and by b) heptamethine cyanine dimerization.

Cyanine truncation is known to proceed both photochemically^[^ ^74^, ^75^ ^–^ ^76^ ^]^ and thermally.^[^ ^77^ ^]^ Specifically via photochemical truncation, a heptamethine cyanine yields a pentamethine cyanine^[^ ^76^ ^]^ and a pentamethine cyanine yields a trimethine cyanine.^[^ ^75^ ^]^ During this process, a two‐carbon unit, consisting of terminal (*ω*‐) and neighboring methine groups, is extruded while the remaining methine groups and their substituents are preserved in the new, truncated cyanine (**Scheme** **9**a). The remaining substituents, however, do not retain their original positions (Scheme 9a).^[^ ^78^ ^]^

In contrast to cyanine truncation, photoinduced dimerization has also been observed in nonsubstituted heptamethine cyanines. During dimerization, two nonsubstituted heptamethine chains are interconnected, yielding a nonamethine cyanine (the longest conjugated polymethine in the dimer) bearing two substituents (Scheme 9b). Unlike truncation, this dimerization is accompanied by bathochromically shifted absorption and emission bands.^[^ ^79^ ^,^ ^80^ ^]^

## 3 Spectral Properties of Cyanines Substituted on Polymethine Chains

Polymethine chain length coarsely sets the spectral region of absorption and emission bands of cyanines, shifting by 100–150 nm with each additional two methine units (see Introduction; Figure 1b).^[^ ^22^ ^]^ Through their donor–acceptor ability (push–pull effect), auxochromes enable fine‐tuning in the range of ≈±50 nm.^[^ ^28^ ^,^ ^30^ ^,^ ^34^ ^]^ Similarly, substituents on the polymethine chain influence spectral properties of cyanines.

Klán and colleagues^[^ ^40^ ^,^ ^64^ ^,^ ^81^ ^]^ systematically investigated the influence of EWGs and EDGs introduced at the *meso‐*, *α‐,* and *ω*‐positions on the optical and photophysical properties of a library of heptamethine cyanines. Their findings showed that introducing an EWG in *α*‐ and *ω*‐positions hypsochromically shifts absorption and emission maxima in relation to the nonsubstituted parent compound ($\lambda_{abs}^{max}=$ 740 nm, $\lambda_{em}^{max}=$ 769 nm). In the *meso‐*position, in turn, the absorption and emission maxima are shifted bathochromically. For instance, a cyano (EWG) group at the *ω*‐position shifts the absorption band to $\lambda_{abs}^{max}=$ 620 nm^[^ ^64^ ^]^; and at the *meso*‐position, to $\lambda_{abs}^{max}=$ 805 nm.^[^ ^81^ ^]^

EDGs, such as ethoxy and methoxy groups, show the opposite trend. An ethoxy group at the *meso*‐position leads to a hypsochromic shift ($\lambda_{abs}^{max}=$ 693 nm, $\lambda_{em}^{max}=$ 747 nm), while a methoxy group at the *α*‐position causes a bathochromic shift ($\lambda_{abs}^{max}=$ 753 nm, $\lambda_{em}^{max}=$ 782 nm).^[^ ^53^ ^,^ ^81^ ^]^ Despite the shift in absorption and emission bands, the Stokes shifts do not vary with the substitution patterns. Moreover, not only electronic but also steric effects influence the spectral properties of cyanines. In heptamethine cyanines, bulky alkyl substituents (i.e., *i*‐Pr, *t*‐Bu) induce a substantial hypsochromic shift of the main absorption band ($\approx \lambda_{abs}^{max}=$ 500–600 nm) and the appearance of a second band of similar intensity (*λ*_abs_ = 370–410 nm).^[^ ^68^ ^]^

Notwithstanding these changes, the molar absorption coefficients of most heptamethine cyanines bearing chain‐substituents remain relatively stable. As reported by different groups,^[^ ^38^ ^,^ ^40^ ^,^ ^64^ ^,^ ^81^ ^]^ the molar absorption coefficients of all compounds range from 1.4 to 2.9 × 10^5^ mol^–1^ dm^3^ cm^–1^ (in methanol and ethanol), similar to nonsubstituted heptamethine cyanines (e.g., the indole‐heptamethine cyanine has *ε* ≈ 2.4 × 10^5^ mol^–1^ dm^3^ cm^–1^ in methanol),^[^ ^81^ ^]^ but not if the substituents on the polymethine chain, e.g., ketone groups hinder polymethine chain delocalization. These groups always cause a substantial blueshift of both absorption and emission bands and decrease molar absorption coefficients.^[^ ^3^ ^,^ ^48^ ^]^

In fact, the fluorescence intensity of heptamethine and pentamethine cyanines is strongly influenced by substituents on the polymethine chain. In methanol, a methoxy EDG at the *α‐*position of the cyanine chain induces a 16‐fold decrease in the fluorescence emission quantum yield (Ф_f_ = 0.015) in comparison with the nonsubstituted heptamethine cyanine (Ф_f_ = 0.24), while an EWG at this position has only a minimal impact (Ф_f_ = 0.38).^[^ ^81^ ^]^ Conversely, the same substituents at the *meso*‐position in heptamethine cyanine lead to different outcomes. For example, an ethoxy EDG causes little to no change in the fluorescence emission quantum yield (Ф_f_ = 0.12), whereas EWGs, such as a cyano‐group, significantly decrease Ф_f_ by about one order of magnitude (Ф_f_ = 0.016). Lastly, steric hindrance also influences the fluorescence intensity. Case in point, *ω*‐substituted pentamethine and heptamethine cyanines bearing bulky groups have a significantly lower fluorescence quantum yield (Ф_f_ = 0.01) than the nonsubstituted heptamethine cyanine.^[^ ^81^ ^]^

## 4 Applications of Cyanines Facilitated by Substituents on their Polymethine Chains

Cyanine dyes have a wide range of applications, thanks to their strong and tunable absorption and fluorescence. Their emission in the visible to NIR region (heptamethine and nonamethine cyanines) enables deeper tissue penetration, so they are extensively used in fluorescence imaging, including live‐cell and in vivo imaging.^[^ ^22^ ^,^ ^27^ ^,^ ^28^ ^,^ ^82^ ^,^ ^83^ ^]^ In molecular diagnostics, cyanine dyes are commonly applied in nucleic acid labeling^[^ ^84^ ^]^ and DNA microarrays.^[^ ^85^ ^]^ In PDT, they serve as photosensitizers, leveraging their optical properties. Beyond these biomedical applications, cyanine dyes also contribute to solar cell design and optical data storage.^[^ ^86^ ^]^

Cyanine dyes bearing substituents on their polymethine chain have also demonstrated their value in a wide range of applications, e.g., diagnostics and therapeutics, including imaging^[^ ^87^ ^]^ and theranostics^[^ ^32^ ^,^ ^35^ ^,^ ^50^ ^,^ ^88^ ^]^ and PDT.^[^ ^89^, ^90^ ^–^ ^91^ ^]^ But these applications can be facilitated or precluded by the substituents on the polymethine chain, particularly if applied as photoremovable protecting groups (PPGs). Herein, we review cases of substituents on the polymethine chain that facilitate cyanine applications.

### 4.1 Imaging

Heptamethine cyanines are commonly applied as fluorescent tags and imaging agents. Several reviews have covered the scope and limitations of their use in this role, including those of cyanines with a substituted polymethine chain, most often a carbocycle at an *α*‐position and/or a substituent at a *meso*‐position.^[^ ^34^ ^,^ ^83^ ^,^ ^88^ ^,^ ^92^, ^93^ ^–^ ^94^ ^]^ Their relatively large auxochrome end groups not only enhance optical properties but also enable functionalization. Nonamethine cyanines with up to five positions substituted on the polymethine chain, absorption maxima of up to *λ*_abs_(max) = 1072 nm and emission maxima of up to *λ*_em_(max) = 1103 nm have also been introduced as tools for multicolor imaging in NIR‐II (and SWIR) regions.^[^ ^13^ ^]^

For imaging, substituents on polymethine chain may not be critical *per se* but make it possible to introduce a recognition motif. For example, Burgess, Chung, Shi, Henary, and their teams have shown that *meso*‐Cl, *α*,*α*‐cyclohexene analogs, and heptamethine cyanine dyes selectively accumulate in solid tumors,^[^ ^94^, ^95^, ^96^ ^–^ ^97^ ^]^ enabling their visualization and even drug delivery.^[^ ^98^ ^]^ The *meso*‐Cl substituent facilitates this selective targeting by promoting cyanine‐protein (e.g., albumin) interactions, which can be silenced by its exchange with a meso‐phenyl.^[^ ^99^ ^,^ ^100^ ^]^

### 4.2 Chemosensors

**Scheme 10:**
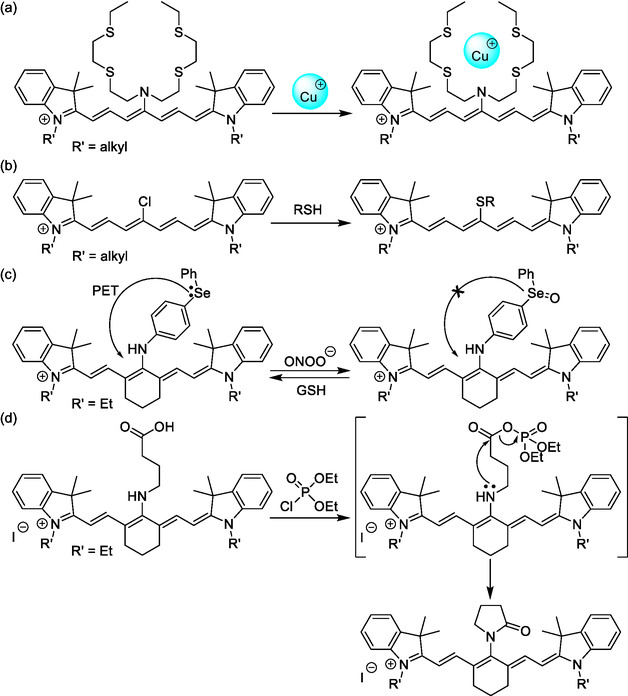
Examples of cyanine chemosensors that detect targets via spectral changes caused by a) ion chelation,^[^ ^102^ ^]^ b) auxochrome modifications,^[^ ^112^ ^]^ c) photoinduced electron transfer (PET) turn‐off triggered by (reversible) oxidation of a key atom in the auxochrome,^[^ ^116^ ^]^ and d) electron donating group (EDG) attenuation in key amino substituents.^[^ ^117^ ^]^

Typically, (chemo‐)sensing by fluorophores relies on changes in their absorption and/or emission properties when they interact with a target molecule or system. To target metal cations, namely Zn^2+^,^[^ ^101^ ^]^ Cu^+^,^[^ ^102^ ^]^ Pd^2+^,^[^ ^103^ ^]^ Hg^2+^,^[^ ^104^ ^]^ MeHg^+^,^[^ ^105^ ^]^ and H^+^,^[^ ^48^ ^,^ ^106^ ^]^ both chelating units and macrocycles have been incorporated at the *meso*‐position of cyanines (e.g., **Scheme** **10**a).^[^ ^30^ ^]^ Cyanines can also act as sensors of i) gastrotransmitters, such as NO,^[^ ^107^ ^]^ H_2_S,^[^ ^108^ ^,^ ^109^ ^]^^1^O_2_,^[^ ^110^ ^]^ H_2_O_2_ and other reactive oxygen species;^[^ ^111^ ^]^ ii) larger and biologically relevant molecules like glutathione^[^ ^112^ ^]^ and enzymes,^[^ ^113^ ^]^ and their activity;^[^ ^114^ ^]^ and iii) highly toxic substances, including nerve agents (mimics).^[^ ^115^ ^]^ Broadly speaking, H_2_S and thiols are detected upon changes in the spectral properties of the cyanine following nucleophilic substitution of the chlorine atom at the *meso*‐position by the corresponding SR group (Scheme 10b). An organoselenium substituent on the cyanine chain can play a dual redox role, sensing both peroxynitrite oxidation and thiol reduction (Scheme 10c).^[^ ^116^ ^]^ Other sensing mechanisms include EDG attenuation of substituents on the polymethine chain^[^ ^117^ ^]^ (Scheme 10d), chromophore destruction/deconjugation, e.g., keto/enol tautomerism of a pH sensor,^[^ ^48^ ^,^ ^108^ ^]^ and a pentamethine CN^–^ sensor based on its addition, effectively isolating one end group.^[^ ^118^ ^]^

### 4.3 Cyanines as PPGs in Targeted Delivery

**Scheme 11:**
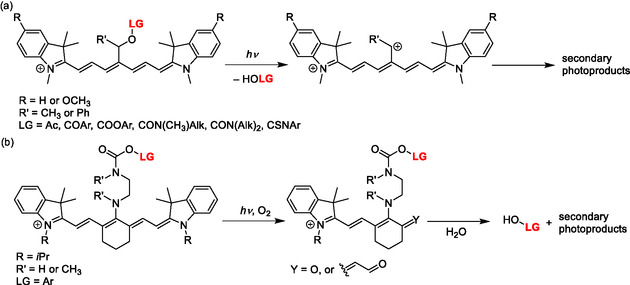
Heptamethine cyanines as PPGs connected to the *meso*‐position through a) –CH(R)– and b) –NRCH_2_CH_2_NR‐COO– linkers.

PPGs and photocages use chromophores that, upon photoactivation, irreversibly release a species with the desired physical, chemical, or biological properties for targeted delivery.^[^ ^119^ ^]^ Among such chromophores,^[^ ^120^ ^]^ heptamethine cyanines stand out for their ability to protect phenols,^[^ ^121^ ^]^ carboxylates, and even gasotransmitters like hydrogen sulfide,^[^ ^122^ ^]^ connected through a –CH_2_– or –CH(R)– substituent on its polymethine chain (at the *meso*‐position) and released upon excitation with a typical quantum yields of the leaving group (LG; molecular cargo) release Ф_release_ in the (1–50) × 10^–5^ range. In combination with the high molar absorption coefficients of heptamethine cyanines, this Ф_release_ results in an uncaging cross‐section *ε*Φ of up to ≈100 mol^–1^ dm^3^ cm^–1^, well‐suited for biological applications,^[^ ^123^ ^]^ and in chemical yields of up to 100%. The absorption and emission of these heptamethine cyanines lie in the red and NIR spectral regions, a property that researchers have sought for decades.^[^ ^31^ ^]^ The mechanism of release of the leaving group depends on the reaction conditions. At low cage (heptamethine‐LG) and oxygen concentrations, the main pathway is direct bond cleavage, yielding an intermediate with a preserved heptamethine cyanine that is subsequently photodegraded (**Scheme** **11**a).^[^ ^124^ ^]^ At normoxia, photooxidation prevails.^[^ ^123^ ^]^

In addition to direct attachment, both carbonates and carbamates can be used as linkers to cage and efficiently release, upon NIR light excitation (*λ*_exc_ ≥ 800 nm), various leaving groups, including etilefrine (heartbeat‐modulating adrenergic agonist) and coumarin (fluorescent tag).^[^ ^123^ ^]^ In some heptamethine cyanines,^[^ ^124^, ^125^ ^–^ ^126^ ^]^ the leaving group is connected through a –CH(R)– linking element (where R = H, alkyl, aryl) to the *meso*‐position, mimicking phenacyl‐ and coumarin‐,^[^ ^127^ ^]^ xanthen‐^[^ ^128^ ^,^ ^129^ ^]^ and BODIPY‐based PPGs,^[^ ^130^ ^]^ all of which release a leaving group through bond heterolysis. In other heptamethine cyanines,^[^ ^121^ ^,^ ^131^ ^]^ LGis also connected to the *meso*‐position, albeit through a –NRCH_2_CH_2_NR‐COO– linker (where R = H, alkyl). LG is then released through a multistep reaction sequence starting from a regioselective photooxidation and concomitant heptamethine cyanine breakdown (Scheme 11b). This strategy has proved effective for LGs such as phenolates, fluorophores,^[^ ^121^ ^]^ and drugs connected through a phenolate motif.^[^ ^132^ ^]^ The upper limit of the LG release quantum yield can be estimated from cyanine photooxidation quantum yields, which were found to be in the range of (5–50) × 10^–4^.^[^ ^133^ ^]^

### 4.4 PDT

Cyanines are used in therapeutic applications, particularly PDT, photothermal therapy (PTT), and drug delivery.^[^ ^29^ ^,^ ^134^ ^,^ ^135^ ^]^ As PDT agents, their efficacy can be increased by introducing substituents to the chain to facilitate singlet oxygen (^1^O_2_) sensitization. For example, pentamethine cyanines with larger aromatic substituents at a *meso*‐position sensitize ^1^O_2_ with high quantum yields (Ф_Δ_ ≈ 0.11).^[^ ^136^ ^]^ In another approach, leveraging the heavy atom effect, heptamethine cyanines with an iodine atom at an *α*‐position (Ф_Δ_ up to 0.25) show > tenfold stronger effect than those with an iodine atom on the end‐group heterocycle (Ф_Δ_ up to ≈0.02).^[^ ^91^ ^]^ Combining both the aryl and the heavy atom effect, Sun et al. introduced a phenylselenyl substituent at the *meso*‐position of a heptamethine cyanine, thus achieving a high ^1^O_2_ emission quantum yield (Ф_Δ_ = 0.11; an 18‐fold increase over the *meso*‐chloro‐cyanine) and inducing cell death in culture models.^[^ ^137^ ^]^ Utilizing symmetry‐breaking, Zhou et al. developed an effective PDT agent in both cell cultures and in vivo by introducing a ketone to the *meso*‐position of heptamethine cyanine.^[^ ^138^ ^]^ Their heptamethine cyanine NIR sensitizer showed a high ^1^O_2_ quantum yield (Ф_Δ_ = 0.34, CH_2_Cl_2_), resulting from molecular vibrational torsion‐enhanced spin–orbit coupling.^[^ ^139^ ^]^

## 5 Summary and Outlook

Research on cyanine dyes has focused on modifications of their terminal auxochromes, while the role of substituents on their polymethine backbone has been overlooked. Here, we reviewed evidence that these cyanine dyes are synthetically available and that substituents on their polymethine chain can significantly influence their photophysical properties, facilitating applications in imaging, diagnostics, and phototherapy. Therefore, leveraging these substituents on the polymethine chain presents a promising path for innovation in cyanine dye chemistry.

## Conflict of Interest

The authors declare no conflict of interest.

## Author Contributions

All authors participated in writing original draft and following iterations. **Peter Šebej** conceived the idea and recruited financial support. All authors approved the final version.
